# Investigation on the Selenization Treatment of Kesterite Cu_2_Mg_0.2_Zn_0.8_Sn(S,Se)_4_ Films for Solar Cell

**DOI:** 10.3390/nano9070946

**Published:** 2019-06-29

**Authors:** Dongyue Jiang, Yu Zhang, Yingrui Sui, Wenjie He, Zhanwu Wang, Lili Yang, Fengyou Wang, Bin Yao

**Affiliations:** 1 Key Laboratory of Functional Materials Physics and Chemistry of the Ministry of Education, Jilin Normal University, Jilin 136000, China; 2 State Key Laboratory of Superhard Materials and College of Physics, Jilin University, Jilin 130012, China

**Keywords:** CMZTSSe films, sol–gel, electrical properties, optical properties, selenization treatment, solar cells

## Abstract

High-selenium Cu_2_Mg_0.2_Zn_0.8_Sn(S,Se)_4_ (CMZTSSe) films were prepared on a soda lime glass substrate using the sol–gel spin coating method, followed by selenization treatment. In this work, we investigated the effects of selenization temperature and selenization time on the crystal quality, and electrical and optical properties of CMZTSSe films. The study on the micro-structure by XRD, Raman, X-ray photoelectron spectroscopy (XPS), and energy-dispersive X-ray spectroscopy (EDS) analysis showed that all CMZTSSe samples had kesterite crystalline structure. In addition, the crystalline quality of CMZTSSe is improved and larger Se takes the site of S in CMZTSSe with the increase of selenization temperature and selenization time. When increasing the selenization temperature from 500 to 530 °C and increasing the annealing time from 10 to 15 min, the morphological studies showed that the microstructures of the films were dense and void-free. When further increasing the temperature and time, the crystalline quality of the films began to deteriorate. In addition, the bandgaps of CMZTSSe are tuned from 1.06 to 0.93 eV through adjusting the selenization conditions. When CMZTSSe samples are annealed at 530 °C for 15 min under Se atmosphere, the crystal quality and optical–electrical characteristics of CMZTSSe will be optimal, and the grain size and carrier concentration reach maximums of 1.5–2.5 μm and 6.47 × 10^18^ cm^−3^.

## 1. Introduction

Thin-film photovoltaic cells have generated enormous attention since the reliable efficiencies of CuInGaSe_2_ (CIGSe) and CdTe have exceeded 20% [1,2,3,4]. While there are numerous benefits of CIGSe and CdTe photovoltaic cells, such as consuming less material and high efficiency, the constituent elements of the materials inevitably have high cost and toxicity. Compared with CIGSe and CdTe, the Cu_2_ZnSn(S,Se)_4_ (CZTSSe) compound has a high absorption coefficient and adjustable bandgaps, and the constituent elements are inexpensive and environmentally friendly [5,6]. Hence, CZTSSe is considered to be a potential absorber material. However, the conversion efficiency of CZTSSe photovoltaic cells hasonly achieved 12.6%, which is still far lower than the conversion efficiency of CIGSe-based solar cells (22.9%) [7,8,9]. In order to improve conversion efficiency and make CZTSSe industrially viable, a lot of researches are still needed. Recently, studies have shown that enhancing the open-circuit voltage (V_oc_) and improving the crystal quality and optical–electrical characteristics of the CZTSSe layer are the main challenges that CZTSSe photovoltaic cells must face [10,11,12]. Considerable researches have been carried out to explore the reason of the lower V_oc_. It was found that the reasons are varied, and one of the frequently mentioned ones is the unsuitable conduction band offset (CBO) [10,11,12]. The CBO of CdS/CZTSSe is affected by the bandgaps of CZTSSe. Therefore, engineering an adjustable bandgap is a practical method to break through the current limit of V_oc_. In order to achieve the goal of tuning CZTSSe bandgaps, a large number of experiments have been carried out [13,14,15,16,17,18].

Recent studies show that adjusting the bandgaps of CZTSSe by partial metal cation replacement is probably an effective approach. Among them, taking the place of Zn with Mg can adapt the bandgaps and enhance the crystallinity of CZTSSe films. In our previous studies, the Cu_2_Mg_x_Zn_x_Sn(S,Se)_4_ samples with different Mg concentrations were successfully synthesized by the sol–gel method [19]. It was found that the bandgaps of Cu_2_Mg_x_Zn_x_Sn(S,Se)_4_ samples can be tuned in the ranges of 1.12 to 0.88 eV as Mg concentration varied from x = 0 to 0.6 [19]. In addition, we investigated the effects of Mg content on the properties of Cu_2_Mg_x_Zn_x_Sn(S,Se)_4_ films in detail. The results of the study showed that the Cu_2_Mg_x_Zn_x_Sn(S,Se)_4_ films with adjusted bandgaps, high crystallinity, and high carrier concentration will be a potential high-efficiency photovoltaic cell absorber material [19]. Furthermore, the realization of bandgap regulation by Mg instead of Zn has the following advantages: Firstly, compared to Cd and Ge, the Mg element is more abundant, low cost, and environmentally friendly [20]. Moreover, the formation of other binary and ternary phases may be eliminated or reduced in the process of synthesizing Cu_2_Mg_x_Zn_x_Sn(S,Se)_4_ films, because ZnS and ZnSe are present, while MgS and MgSe are unstable in the process of synthesizing the precursor solution [20]. Therefore, it is concluded that Cu_2_Mg_x_Zn_x_Sn(S,Se)_4_ is worthy of study as a potential absorbing layer material.

As we all know, for the sake of improving the properties of CZTSSe and obtaining CZTSSe films with a single phase and large crystal size, a heat treatment of the precursor films at an elevated temperature (>500 °C) is usually required [21,22]. A large number of studies have shown that the selenization temperature and selenization time significantly influence the properties of the films, including the crystal quality, and optical and electrical properties [23,24,25]. The influence of selenization treatment on the physical performance of CZTSSe has been investigated by extensive researches [21,22,23,24,25]. However, the effects of selenization temperature and selenization time on the phase evolutions, crystal quality, and optical–electrical characteristics of Cu_2_Mg_x_Zn_x_Sn(S,Se)_4_ have not been reported so far. Hence, in the present work, Cu_2_Mg_0.2_Zn_0.8_Sn(S,Se)_4_ (CMZTSSe) samples annealed at different selenization conditions are synthesized, and the influences of selenization temperature and selenization time on the structure, and optical and electrical properties of CMZTSSe samples are investigated in detail.

## 2. Experimental Methods

### 2.1. Synthesis of Cu_2_Mg_0.2_Zn_0.8_Sn(S,Se)_4_ Precursor Films

The CMZTSSe precursor films were synthesized in two steps. The first process was to prepare the CMZTSSe precursor solution by a simple and convenient sol–gel technique. We dissolved Cu(CH_3_COO)_2_·H_2_O (0.8086 g), MgCl_2_·6H_2_O (0.1787 g), Zn(CH_3_COO)_2_·2H_2_O (0.4794 g), SnCl_2_·2H_2_O (0.5077 g), and thiourea (1.3702 g) into 2-methoxyethanol (10 mL), and stirred for 10–15 min at room temperature. The monoethanolamine (MEA) (0.2 mL) was added to the precursor solutions at the end. During the precursor solution preparation process, in order to obtain high-quality CMZTSSe films, the ratio of Cu/(Mg + Zn + Sn) was 0.82 and the ratio of (Zn + Mg)/Sn was 1.2. In our previous studies, we found that the surface morphology, and optical and electrical properties of Cu_2_Mg_x_Zn_x_Sn(S,Se)_4_ films were optimal when the proportion of Mg/(Mg + Zn) was 0.2 [17]. Therefore, in the present work, the proportion of Mg/(Mg + Zn) was set to 0.2. Then, in order to dissolve the raw materials completely and obtain the CMZTSSe sol–gel solution at room temperature, we stirred the solution until the precursor solution color became colorless and transparent. The second procedure was to obtain the CMZTSSe precursor films through the spin coating method. We spun the precursor solution of CMZTSSe at 3000 r for 30 s, followed by drying at 300 °C for 5 min in air. In order to acquire CMZTSSe films with micrometer thicknesses, the process of the coating and drying was repeated several times.

### 2.2. Selenization of Cu_2_Mg_0.2_Zn_0.8_Sn(S,Se)_4_ Films

For the sake of studying the effect of annealing treatment on the properties of CMZTSSe films, the rapid annealing treatment was implemented for CMZTSSe precursor films at various selenization temperatures and selenization times under the same selenium atmosphere. We used fixed-quality selenium powder (15 mg) to anneal the precursor CMZTSSe films in the selenide annealing furnace, and increased the selenization temperature from 500 to 560 °C, while the selenization time was adjusted in the range of 10–20 min to obtain CMZTSSe samples at varied annealing conditions.

### 2.3. Materials Characterization

The structural characteristics and chemical composition of CMZTSSe were measured using X-ray power diffraction (XRD, Rigaku Corporation, Tokyo, Japan), Raman spectroscopy (Renishaw, London, UK) with a 514 nm laser wavelength, and X-ray photoelectron spectroscopy (XPS, Thermo Fisher Scientific, Waltham, MA, USA) (Al Kα was used as the X-ray source). Scanning electron microscopy (SEM) (Hitachi S-4800, JEOL Ltd., Tokyo, Japan) was performed to study the surface morphology of CMZTSSe films. The energy-dispersive X-ray spectroscopy (EDS, JEOL Ltd., Tokyo, Japan) system was used to analyze elemental content. The optical and electrical performances of CMZTSSe films were characterized by UV-Vis-NIR spectra (UV-3101PC, Tokyo, Japan) and room temperature Hall measurement, respectively.

## 3. Results and Discussion

### 3.1. Influence of Annealing Temperature on the Properties of Cu_2_Mg_0.2_Zn_0.8_Sn(S,Se)_4_ Films

As we all know, the properties of absorbers are easily affected by annealing temperature. For the sake of studying the effect of selenization temperature on the microstructure and photoelectric characteristics of CMZTSSe films during the selenization process, the CMZTSSe samples were annealed for 15 min under Se ambience at different temperatures of 500, 530, and 560 °C, hereafter named as A1, A2, and A3, respectively. In addition, the CZTSSe film annealed at a temperature of 530 °C for 15 min will be used as a reference, named as sample A.

**Figure 1:**
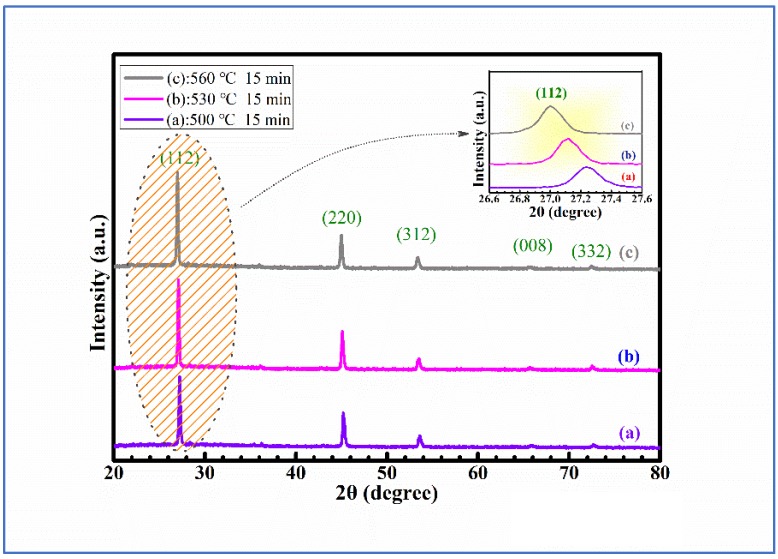
XRD spectra of Cu_2_Mg_0.2_Zn_0.8_Sn(S,Se)_4_ (CMZTSSe) films annealed at different temperatures (**a**) 500 °C, (**b**) 530 °C, (**c**) 560 °C. Inset: Enlarged view of the corresponding (112) diffraction peak of the CMZTSSe films annealed at different temperatures.

XRD spectra were always used to evaluate the crystal quality and assess the probable impurity phase during the selenization process. Figure 1 represents the XRD spectra of CMZTSSe samples annealed at different temperatures from 500 to 560 °C (samples A1, A2, and A3). The CMZTSSe films at all varied annealing temperatures were in the kesterite phase, with peaks corresponding to the (112), (220), (312), (008), and (332) planes of Cu_2_ZnSnS_4_ (CZTS) with the kesterite phase [26,27]. As shown in Figure 1, the characteristic peaks of impurity phases were not found, which indicates that the single-phase CMZTSSe with kesterite structure was formed at all varied annealing temperatures. Figure 1a indicates the XRD patterns for sample A1, annealed at 500 °C under atmosphere of Se, and it was found that the peak intensity is weaker. By increasing the annealing temperature to 530 °C, as shown in Figure 1b, the peak intensity was found to be enhanced, indicating that the crystal quality of sample A2 is enhanced at 530 °C. For sample A3, with the annealing temperature of 560 °C, the peak intensity of XRD only slightly changed, as seen from Figure 1c. As seen from the inset, the dominant characteristic peak (112) is observed to be shifted to smaller angles, from 27.20° to 26.93°, with the annealing temperature increasing from 500 to 560 °C. This is due to the variety in atomic-lattice distance caused by elemental replacement, where the element Se will take the place of S in the CMZTSSe compound with the increase of annealing temperature. According to the results of XRD, there are no secondary phases in CMZTSSe films annealed at different selenization temperatures from 500 to 560 °C.

Table 1 shows the values of full width at half-maximum (FWHM), grain size, the a-axis lattice constant (a), and the c-axis lattice constant (c) for the CMZTSSe films annealed at different temperatures, which are obtained from the (112) peak in the XRD profile. It can be seen from Table 1 that, as the annealing temperature increases from 500 to 560 °C, the grain size increases first and attains a maximum value at 530 °C, and then decreases when the annealing temperature is continuously increased up to 560 °C. An opposite changing trend was observed concurrently for the FWHM—when increasing the annealing temperature up to 530 °C, the FWHM has a minimum value. Meanwhile, the a-axis lattice constant gradually increases from 5.669 to 5.691 Å as the annealing temperature increases from 500 to 560 °C. The c-axis lattice constant also increases from 11.305 to 11.635 Å. The increases in the lattice constants are ascribed to the rise of the Se content in films, where the S (0.184 nm) atoms were replaced by the larger Se (0.198 nm) atoms with the increasing annealing temperature. This may be verified by the EDS results later.

It is well known that the discernment of the secondary phases in CZTSSe compounds by XRD patterns is difficult, owing to the nearly overlapped XRD patterns of the secondary ZnS(Se) and Cu_2_SnS(Se)_3_ with the kesterite CZTSSe [28]. Therefore, Raman spectroscopy measurement is usually applied as an auxiliary technique to detect possible impurity phases, because it is sensitive to lattice vibrations.

**Figure 2:**
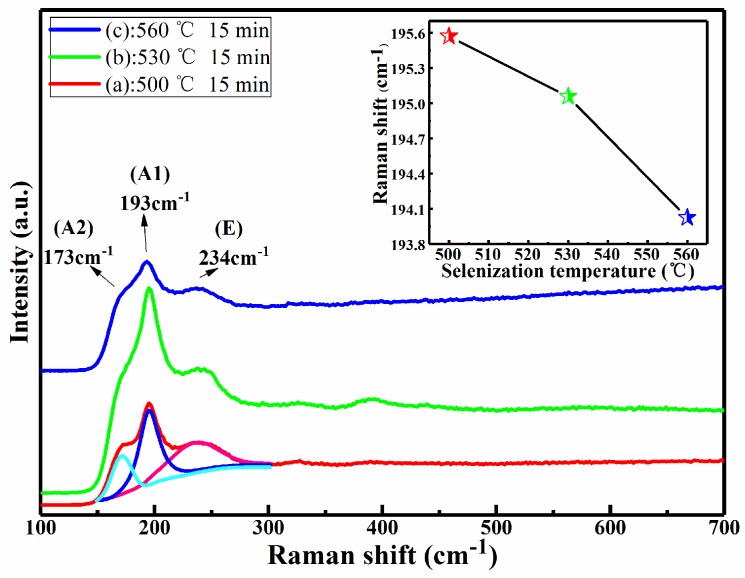
Raman spectra of the CMZTSSe films annealed at different temperatures. Inset: The main Raman peaks of A1 mode for CMZTSSe films annealed at different temperatures.

Figure 2 shows the Raman spectra of CMZTSSe films annealed at different annealing temperatures from 500 to 560 °C (samples A1, A2, and A3). The Raman spectrum of sample A1 was fitted using the Gaussian fitting method, and the peaks at 173, 193, and 234 cm^−1^ can be observed. The peaks at 173 and 193 cm^−1^ conform to the A (A1 and A2) mode Raman vibration peaks of the kesterite CZTSSe phase, as reported in the previous literature [29]. The A modes are pure anion modes which correspond to vibrations of pure chalcogen (S or Se) atoms surrounded by motionless neighboring atoms. The result exhibits a broad peak located at 234 cm^−1^ that corresponds to the E vibration mode in connection with Sn–Se bonding in the kesterite CZTSSe phase [29]. In addition, it can be seen that the Raman peaks of all samples conform with the vibration peaks of the kesterite CZTSSe phase. The Raman peaks of the other secondary phases were not discovered. This indicates that all annealed samples consist of a single phase of CZTSSe with kesterite structure. Furthermore, with the variation of annealing temperature from 500 to 560 °C, it was found that all Raman peaks were shifted to the lower values, especially for the A1 mode peak. The inset of Figure 2 displays the tracking of the peak position of the A1 vibration mode, and it is obviously noted that the A1 vibration peak shifted from 195.6 to 193.9 cm^−1^ as the annealing temperature changed from 500 to 560 °C. This phenomenon can be attributed to the increment of selenization temperature from 500 to 560 °C, which easily allows larger Se to replace S in the CZTSSe, significantly increasing the lattice constant. It was found from the Raman results that the secondary phases were not discovered, which is consistent with the XRD results. In addition, the peak shift as observed from the XRD and Raman spectra is considered to be due to larger Se taking the site of smaller S in CMZTSSe films with the increase of selenization temperature from 500 to 560 °C, which will be better understood from the compositional and morphological studies later.

**Figure 3:**
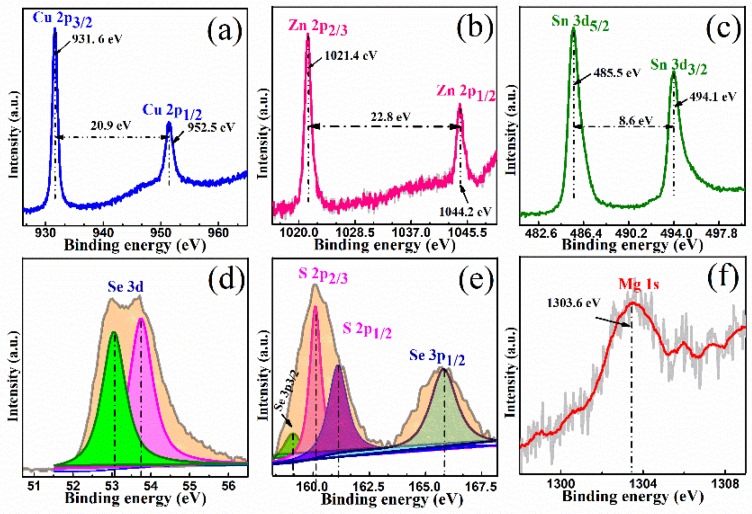
X-ray photoelectron spectroscopy (XPS) spectra of CMZTSSe films annealed at 530 °C for 15 min: (**a**) Cu, (**b**) Zn, (**c**) Sn, (**d**) Se, (**e**) S, and (**f**) Mg.

XPS is sensitive to information about the chemical bonding state and element content. As shown in Figure 3, we identify the elemental composition and valence states of the constituent elements (Cu, Zn, Sn, Se, S, and Mg) in CMZTSSe films annealed at 530 °C for 15 min by XPS measurement, and all peaks were corrected by the C1s binding energy (284.8 eV). As seen from Figure 3a, the Cu 2p XPS spectrum consists of a Cu 2p_3/2_ peak and a Cu 2p_1/2_ peak at 931.6 and 952.5 eV, respectively, with a peak separation of 20.9 eV, which indicates that Cu is in the state of +1 [30]. Figure 3b displays a Zn 2p XPS spectrum, and the peaks presented at 1021.4 and 1044.2 eV are ascribed to Zn 2p_3/2_ and Zn 2p_1/2_, respectively. The binding energy interval between the Zn 2p_3/2_ peak and the Zn 2p_1/2_ peak is 22.8 eV, indicating the existence of divalent Zn ions [31]. Figure 3c shows the XPS spectrum of Sn 3d, and two peaks attributed to Sn 3d_5/2_ and Sn 3d_3/2_ can be observed at 485.5 and 494.1 eV. The energy difference of the two Sn 3d peaks is 8.6 eV, suggesting the presence of the Sn^4+^ state [32]. Figure 3d displays the Se 3d XPS spectrum, and the S 2p high-resolution spectrum is shown in Figure 3e. The Se 3d XPS spectra can be fitted into two sub-peaks located at 53.3 and 53.8 eV, which can be ascribed to Se 3d_3/2_ (green area) and Se 3d_1/2_ (purple area), respectively. The above results indicate that Se in the films is likely to exist in the Se^2−^ state [33]. It is well known that the S 2p core level and Se 3p core level are almost overlapping, and we used the Gaussian fitting method to fit the XPS spectra into four sub-peaks presented at 159.1, 160.2, 161.3, and 165.8 eV, which are ascribed to Se 2p_3/2_ (light green area), S 2p_3/2_ (pink area), S 2p_1/2_ (deep purple area), and Se 3p_1/2_ (grey green area), respectively. The S 2p_3/2_ and S 2p_1/2_ peaks located at 160.2 and 161.3 eV, respectively, are in the standard reference value range (160–164 eV) [33], which means that S exists in the form of S^2−^. Figure 3f displays the XPS high-resolution spectrum of Mg 1s, and the peak can be observed at 1303.6 eV, which indicates that divalent Mg^2+^ exists in our study [19]. The results of the XPS analysis show that the constituent elements (Cu, Zn, Sn, Se, S, and Mg) exist in the forms of Cu^1+^, Zn^2+^, Mg^2+^, Sn^4+^, Se^2−^, and S^2−^ in CMZTSSe.

**Figure 4:**
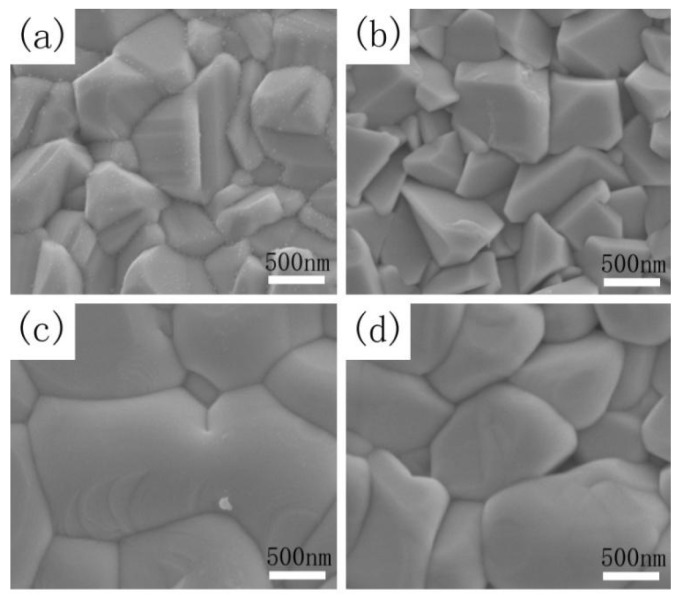
SEM images of CZTSSe annealed at (**a**) 530 °C and CMZTSSe films annealed at (**b**) 500; (**c**) 530; and (**d**) 560 °C.

Figure 4a–d shows the scanning electron microscopy (SEM) images of the CZTSSe film annealed at 530 °C for 15 min (sample A) and the CMZTSSe films annealed under atmosphere of Se for 15 min at temperatures of 500, 530, and 560 °C (samples A1, A2, and A3). Figure 4a shows the surface SEM images of sample A. As we can see from Figure 4a, irregular and small grain sizes of 0.5–0.8 µm were observed. In addition, it was clearly seen that the surface morphology of Cu_2_ZnSn(S,Se)_4_ films is very rough. Figure 4b shows the SEM of sample A1, and it is clearly seen that the film consists of nanograins, with the grain size being even smaller than that of sample A, and the surface morphology is also rough. As shown in Figure 4c, the crystal quality was improved for sample A2, and the grain size of the film reached 1.0–2.5 µm while the surface morphology became smooth and compact. When the selenization temperature was increased to 560 °C, the grain size of sample A3 slightly reduced to 1.0–1.5 µm and displayed a rough morphology, as displayed in Figure 4d. The results indicate that the proper selenization temperature is 530 °C, and that this is beneficial to promote the grain growth. Excessively high or low temperatures will cause the deterioration of film quality. When the selenization temperature is 530 °C, not only does the grain size of CMZTSSe film reach its maximum, but the surface morphology of the film also becomes smooth and compact.

**Figure 5:**
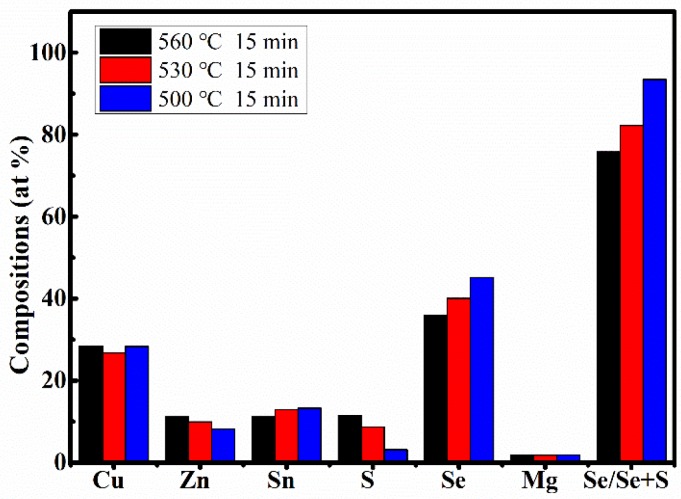
Energy-dispersive X-ray spectroscopy (EDS) composition analyses of CMZTSSe films annealed at different temperatures.

As we all know, the photoelectric properties of CZTSSe-based films need to rely heavily on the stoichiometric ratios of Cu, Zn, Sn, S, and Se in CZTSSe-based films [34]. Table 2 displays the EDS results of CMZTSSe films annealed at different temperatures from 500 to 560 °C (samples A1, A2, and A3). According to the EDS results, we confirmed the existence of Cu, Zn, Mg, Sn, S, and Se elements in samples A1, A2, and A3. It was found that the atomic percentages of Se increased from 35.87% to 45.20% and S evidently decreased from 11.46% to 3.16% with increasing selenization temperature from 500 to 560 °C, indicating that Se will partially replace S in the CMZTSSe compound. The compositions of the other elements (Cu, Sn, and Mg) in all three samples were found to be nearly the same, while the atomic percentages of Zn decreased from 11.30% to 8.16%, indicating that Zn loss happened when the selenization temperature changed from 500 to 560 °C. In the precursors, the ratios of Cu/(Zn + Mg + Sn) and Mg/(Mg + Zn) were about 0.82 and 0.2, respectively. The ratios of Mg/(Mg + Zn) in all the films were close to 0.2, but the ratios of Cu/(Zn + Mg + Sn) in all films became significantly larger. This may be due to the decrease of Zn content as the annealing temperature increases. As mentioned before, Se/(S + Se) > 50% is highly suitable for the fabrication of high-efficiency solar cells [35]. The percentage of Se/(S + Se) is 75.79% for A1, and the percentages of Se/(S + Se) increase to 82.22% and 93.47% for A2 and A3, respectively, indicating that A1, A2, and A3 samples are suitable for the fabrication of efficient solar cell devices. Sample A2 has an appropriate Se/(S + Se) ratio and the grain size became larger compared to samples A1 and A3. Therefore, sample A2 is more suitable to fabricate the high-efficiency solar cells. Figure 5 summarizes the elements composition analysis of CMZTSSe films according to Table 2. As shown from Figure 5, when increasing the selenization temperature from 500 to 560 °C, the proportion of Se increases gradually while the content of S decreases, while the atomic percentages of Cu, Zn, Sn, and Mg remain relatively constant compared with those of S and Se.

**Figure 6:**
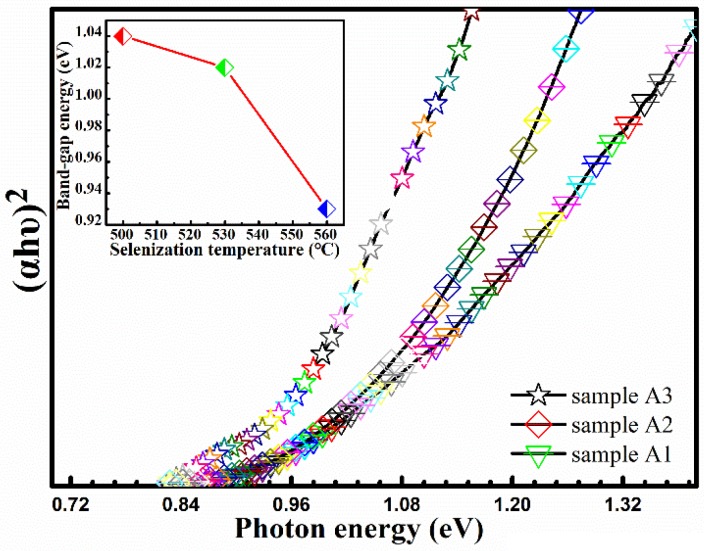
The plot of (*αhυ*)^2^ vs. *hυ* for the absorption spectra. Inset: Bandgap variation as a function of the selenization temperature.

In order to research the influence of selenization temperature on the optical bandgaps of CMZTSSe films (samples A1, A2, and A3), we studied the optical absorption measurements of the CMZTSSe films annealed at different selenization temperatures by an UV-vis-NIR spectrophotometer. Figure 6 displays the (*αhυ*)^2^–*hυ* plots of CMZTSSe films. We use the solids band theory to express the relation between the absorption coefficient (*α*) and the photon energy (*hυ*) as follows [36]:

(*αhυ*) = *B*(*hυ* − *E*_g_)*^n^* (1)

where *h*, *B*, *υ*, and *E*_g_ are Plank’s constant, a constant, photon frequency, and optical bandgap, respectively. The values of n can employ 3, 2, 3/2, and 1/2, when transitions are indirect unallowed, indirect allowed, direct unallowed, and direct allowed, respectively [37]. The values of n can employ 1/2 for direct bandgaps of semiconductor CZTSSe [36]. By using the Equation (1) and the data in Figure 6, the bandgaps of CMZTSSe are evaluated to be 1.04, 1.02, and 0.93 eV for samples A1, A2, and A3, respectively, as shown in the illustration of Figure 6. It was found that the bandgap values of samples A1, A2, and A3 gradually decrease with increasing selenization temperature, which can be attributed to the changes of crystal lattice and disparities in electronegativities owing to alloying and modified atomic structures through Se taking the site of S.

The electrical properties of the absorbing layer are also important factors affecting the efficiency of solar cells. Table 3 displays the electrical characteristics of CMZTSSe annealed at different temperatures (samples A1, A2, and A3) by the Vander Paw method at room temperature. It was found that the CMZTSSe films annealed at different temperatures behave with p-type semiconductor characteristics. When the selenization temperature is increased from 500 to 530 °C, the resistivity first decreases from 6.18 × 10^0^ to 2.85 × 10^−1^ Ω·cm, then increases to 1.10 × 10^2^ Ω·cm at the selenization temperature of 560 °C. Obviously, when the selenization temperature is 530 °C, the resistivity is optimal. Simultaneously, it is clear that the corresponding carrier concentration shows a best value of 6.47 × 10^18^ cm^−3^ at the selenization temperature of 530 °C. In addition, the mobility reduced from 1.09 × 10^0^ cm^2^V^−1^s^−1^ (500 °C) to 3.31 × 10^−1^ cm^2^V^−1^s^−1^ (530 °C), but increased to 1.04 × 10^−1^ cm^2^V^−1^s^−1^ at the selenization temperature of 560 °C. We analyzed the reasons for the change of CMZTSSe electrical properties, combined with the characterization of SEM. It was concluded that the defects at the surfaces of the absorbing layers are passivated, and owing to the crystal quality of CMZTSSe films improving with the selenization temperature increasing from 500 to 530 °C, the resistivity and carrier concentration achieve the best values with sample A2. It is obvious that the deterioration of resistivity and carrier concentration is owing to the deterioration of crystal quality when the selenization temperature further increases from 530 to 560 °C.

### 3.2. Effect of Selenization Time on Properties of CMZTSSe Films

After a series of analyses and characterizations, it was proved that the best selenization temperature is 530 °C. As we all know, selenization time is also one of the major parameters influencing the performance of the absorber layer. In our study, after optimizing the annealing temperature, the influence of annealing time on the properties of CMZTSSe films has been studied. The CMZTSSe films were annealed for 10, 15, and 20 min at 530 °C under atmosphere of Se, afterward referred as B1, B2, and B3, respectively.

**Figure 7:**
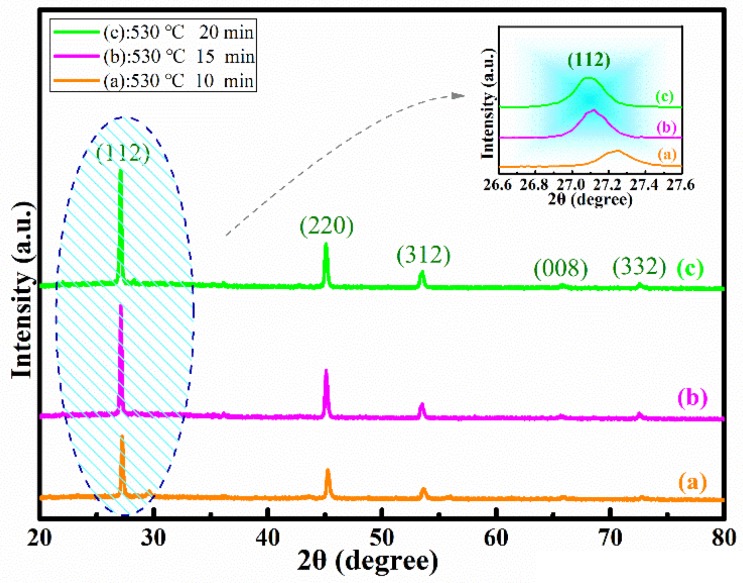
XRD spectra of CMZTSSe films annealed at 530 °C for different time (**a**) 10 min, (**b**) 15 min, (**c**) 20 min. Inset: Enlarged view of the corresponding (112) diffraction peaks of the CMZTSSe films annealed for different times.

Figure 7a–c shows the XRD patterns of CMZTSSe films annealed for different times from 10 to 20 min (samples B1, B2, and B3). For the samples B1, B2, and B3, five diffraction peaks located at 28.53°, 47.33°, 56.17°, 69.27°, and 76.44° were observed, conforming to the (112), (220), (312), (008), and (332) planes of kesterite CZTS respectively [25,26]. The characteristic diffraction peaks of other impurity phases were not observed in Figure 7. We can explore the influence of annealing time on the structural performance of CMZTSSe films by observing the peak intensity and peak shift. It is observed from Figure 7a,b that the intensity of the (112) peak is increased, which indicates that the crystal quality is enhanced by increasing the annealing time from 10 to 15 min. Furthermore, the characteristic peak intensity is almost unchanged with the increase of annealing time from 15 to 20 min, as displayed in Figure 7c. In addition, the position of the (112) peaks are shifted to lower 2θ angle with selenization time increasing, as shown in the illustration of Figure 7. Since the concentration of Se in the CMZTSSe matrix increases with the increase of selenization time, there is enlargement of unit cell size, causing the change of the lattice distance in the films. According to the analysis results of XRD, it was found that the crystal structure of CMZTSSe was not changed with the increase of annealing time, and we speculate that the crystal growth of CMZTSSe was completed when the annealing time reached 15 min.

The full width at half-maximum (FWHM), grain size, a-axis lattice constant (a), and c-axis lattice constant (c) of the films annealed for different times from 10 to 20 min (samples B1, B2, and B3) are displayed in Table 4. It was found that by increasing the annealing time from 10 to 20 min, the grain size first increases and then decreases, while the FWHM value first decreases and then increases. When the annealing time is 15 min, the grain size has a maximum value, while the FWHM has a minimum value. The a-axis lattice constant gradually increases from 5.665 to 5.684 Å as the annealing time increases from 10 to 20 min. Meanwhile, the c-axis lattice constant also increases from 11.334 to 11.453 Å. It was concluded that the optimal annealing time is 15 min.

**Figure 8:**
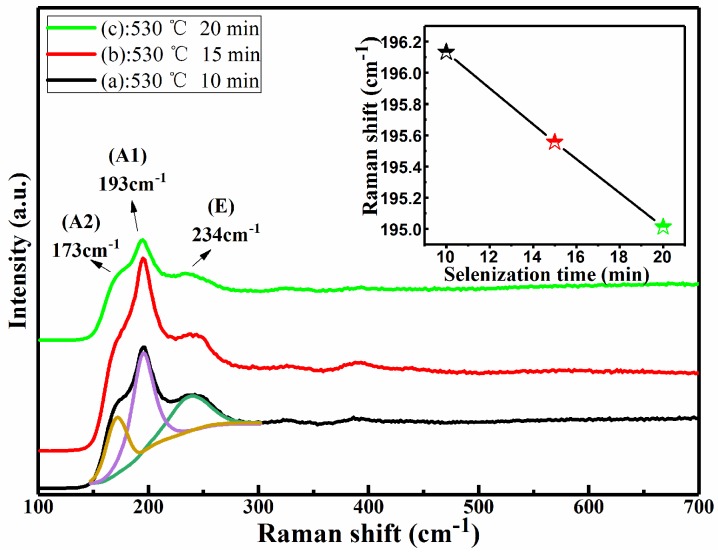
Raman spectra of the CMZTSSe films annealed for different times. Inset: The main Raman peaks of A1 mode for CMZTSSe films annealed for different times.

As we all know, the diffraction peaks of Cu_3_SnS_4_ with a tetragonal structure and ZnS with a cubic structure are close to the diffraction peaks of CZTSSe with a kesterite structure [38,39]. Thus, it is very difficult to detect possible secondary phases by XRD only. In order to further detect the phase compositions of CMZTSSe films, Raman scattering spectra were measured for the films annealed for different times from 10 to 20 min (samples B1, B2, and B3), as shown in Figure 8. The Raman spectrum of sample B1 was fitted using the Gaussian fitting method, and three Raman peaks located at ~193 (A1 mode), 173 (A2 mode), and 234 cm^−1^ (E mode) were observed, which conform to the Raman characteristic peaks of CZTSSe [29]. The characteristic peaks of some possible impurity phases were not detected. It is suggested that the CMZTSSe films are composed of a single phase of kesterite CZTSSe. In addition, these characteristic peaks slightly shift to lower values as the selenization time increases, owing to the incorporation of Se in the CMZTSSe compound. It should be noted that the Raman spectra are consistent with the XRD patterns, and some possible secondary phases (Cu_2_SnSe_3_, SnSe_2_, SnSe, ZnSe, and MgSe) were observed. Furthermore, the phase change of the CMZTSSe films did not occur with increasing selenization time from 10 to 20 min. It was concluded that the pure-phase CMZTSSe films were successfully synthesized.

**Figure 9:**
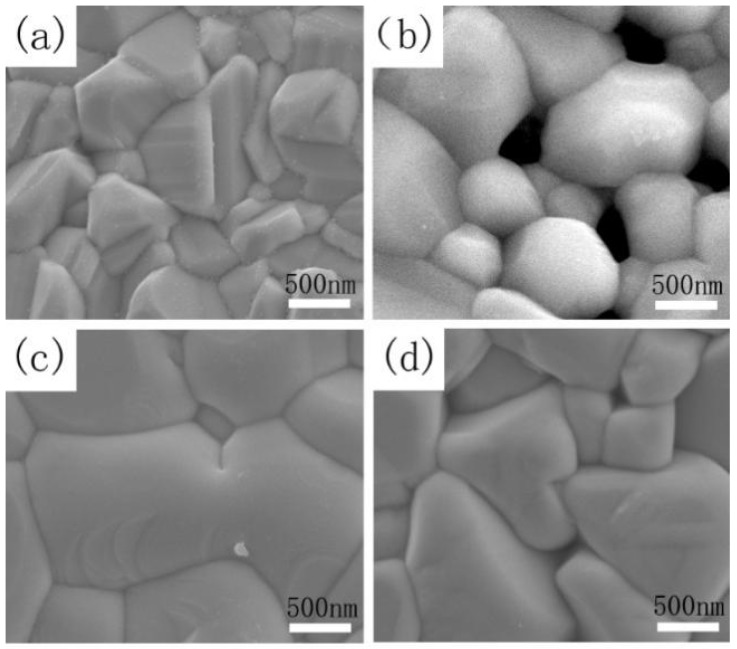
SEM images of CZTSSe annealed at 530 °C for (**a**) 15 min and CMZTSSe films annealed at 530 °C for (**b**) 10 min; (**c**) 15 min; and (**d**) 20 min.

Figure 9a–d depicts the SEM surface images of CZTSSe annealed at 530 °C for 15 min (sample A) and CMZTSSe films annealed at 530 °C for different times from 10 to 20 min (samples B1, B2, and B3), respectively. As shown in Figure 9a, the surface of the sample A displays pin-hole free morphology and small grain size between 500–800 nm, with a rough surface. Figure 9b shows the surface morphology of sample B1, and it was clearly observed that the CMZTSSe film still consists of nanograins, where the grain size is between 500 and 1000 nm with a large number of holes on the surface. As the annealing time increased to 15 min, obvious morphological change is observed (Figure 9c), with the grains size of sample B2 increasing sharply to the micron level (1.0–2.5 μm) and the surface becoming dense and flat. By further extending the annealing time to 20 min, the surface morphology of sample B3 becomes rough but still dense, and the grains size slightly decreases to 0.8–1.3 μm. It was concluded that the optimal selenization time is 15 min, and the CMZTSSe films obtained at this time have the best crystallinity, the largest crystal grain size, and their surfaces are dense and flat.

**Figure 10:**
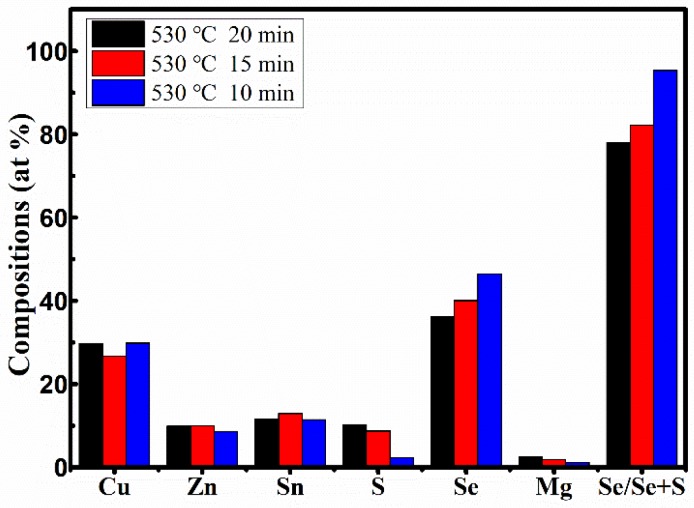
EDS composition analyses of CMZTSSe films annealed for different times.

The EDS results of the films annealed for different times from 10 to 20 min (samples B1, B2, and B3) are displayed in Table 5. It was found that by increasing the annealing time from 10 to 20 min, the atomic percentage of S evidently decreases from 10.23% to 2.26%, the atomic percentage of Se increases from 36.24% to 46.44%, and the ratio of Se/(Se + S) significant increases from 77.99% to 95.36%. The ratios of Cu/(Zn + Mg + Sn) in all films were significantly larger than those in the precursor, and the ratios of Mg/(Mg + Zn) in all films significantly decreased from 0.19 to 0.12 with increasing annealing time from 10 to 20 min. These changes were ascribed the decrease of Mg content with the increase of the annealing time, as shown in Table 5. Figure 10 represents the elemental composition analysis of the films annealed for different times from 10 to 20 min. It can be clearly seen that the atomic percentages of Se increase, while the atomic percentages of S decrease with the increase of annealing time. Compared with the increase of Se content and the decrease of S content, the atomic percentages of other elements (Cu, Zn, and Sn) changed only slightly. Furthermore, the changes were irregular and almost negligible, and the changes had less effect on the crystal quality of CMZTSSe films. According to the analysis of elemental composition, the change of selenization time mainly affects the atomic percentages of Se and S elements, and has little effect on other elements.

**Figure 11:**
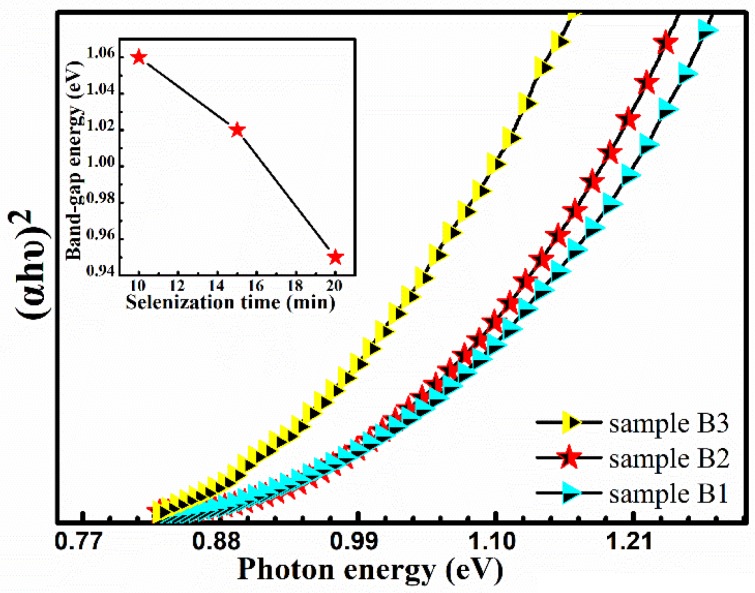
The plot of (*αhυ*)^2^ vs. *hυ* for the absorption spectra. Inset: Bandgap variation as a function of the selenization time.

Microstructure, composition, and grain size have great influences on the crystal quality of CMZTSSe, and the bandgaps are also crucial. According to the previous analysis, the change in the ratio of Se/(Se + S) with the increase of selenization time significantly influences the crystal quality, composition, and grain size. The effect of annealing time on the optical bandgaps of the CMZTSSe films has been evaluated by a UV-vis-NIR spectrophotometer. The theoretical basis of bandgap calculation is consistent with Formula 1. As shown in Figure 11, the bandgaps of the CMZTSSe films show a declining trend (1.06–0.95 eV) with increasing annealing time from 10 to 20 min. The illustration of Figure 11 displays the dependence of bandgaps on selenization time for CMZTSSe films. We can clearly see that the values of bandgap are 1.06, 1.02, and 0.95 for sample B1, sample B2, and sample B3, respectively. Combined with the analysis results of XRD, Raman, and EDS, the decline in bandgaps with the increase of the selenization time is ascribed to the increase of elemental Se.

As shown in Table 6, the impacts of selenization time on the conductivity, carrier concentration, and mobility of CMZTSSe (samples B1, B2, and B3) were investigated by Hall measurements at room temperature. It was observed that the p-type conductivity of CMZTSSe was not changed with the increase of annealing time from 10 to 20 min. As shown in Table 6, when the annealing time increased from 10 to 15 min, the hole concentration of the CMZTSSe films increased obviously from 9.22 × 10^17^ to 6.47 × 10^18^ cm^−3^, the resistivity decreased from 6.18 × 10^0^ to 2.85 × 10^−1^ Ω·cm, and the mobility decreased from 1.09 × 10^0^ to 3.31 × 10^−1^ cm^2^V^−1^s^−1^. Combined with the analysis results of SEM, by increasing annealing time from 10 to 15 min, the grain size of CMZTSSe becomes bigger and the surface becomes smooth and hole-free, which leads to the improvement of electrical performance. When the annealing time increases from 15 to 20 min, the crystallinity of CMZTSSe films deteriorates, and hence the hole concentration and resistivity decrease to 5.54 × 10^17^ cm^−3^ and 1.10 × 10^2^ Ω·cm, respectively. It was found that when the selenization temperature and selenization time are 530 °C and 10 min, the best electrical properties of the films are obtained.

## 4. Conclusions

In summary, we have successfully fabricated pure-phase CMZTSSe films at different selenization temperatures and selenization times through the sol–gel method. It was found that the properties of CMZTSSe films are greatly affected by selenization temperature and selenization time. Combined with the results of XRD, Raman, XPS, and EDS, it is clear that single-phase CMZTSSe films have been synthesized at different selenization temperatures and times, and the content of Se increases gradually while the content of S decreases gradually with increasing selenization temperature and selenization time. The SEM results suggested that the crystal quality of CMZTSSe is the best at the optimal selenization condition of 530 °C for 15 min, where the grain size reaches 1.0–2.5 μm. In addition, the grain-boundary passivation due to the crystal quality improvement will result in the improvement of electrical performance. The CMZTSSe films with p-type conductivity and high hole concentration of 6.47 × 10^18^ cm^−3^ were obtained by selenization at 530 °C for 15 min. The *E*_g_ of CMZTSSe films is decreased from 1.04 to 0.93 eV with increasing selenization temperature from 500 to 560 °C. When selenization time is increased from 10 to 20 min, the *E*_g_ of CMZTSSe can be adjusted from 1.06 to 0.96 eV. It is concluded that the structure, and optical and electrical properties of CMZTSSe will be optimal at an optimized selenization temperature and selenization time of 530 °C and 15 min, respectively, which will create an ideal absorber material for preparing higher efficiency kesterite solar cells.

## Figures and Tables

**Table 1:** The full width at half-maximum (FWHM), grain size, a-axis lattice constant (a), and c-axis lattice constant (c) for CMZTSSe films annealed at different temperatures.

Sample	Temperature (°C)	Time (min)	a (Å)	c (Å)	Crystalline Size (nm)	FWHM
A1	500	15	5.669	11.305	58.2	0.172
A2	530	15	5.681	11.415	71.1	0.156
A3	560	15	5.691	11.635	67.1	0.163

**Table 2:** EDS results of the CMZTSSe films annealed at different temperatures from 500 to 560 °C.

Sample	Temperature (°C)	Cu (at%)	Zn (at%)	Sn (at%)	Mg (at%)	S (at%)	Se (at%)	Se/(S + Se)	Cu/(Zn + Mg + Sn)	Mg/(Mg + Zn)
A1	500	28.46	11.30	11.14	1.78	11.46	35.87	75.79	1.18	0.14
A2	530	26.71	9.91	12.88	1.76	8.67	40.08	82.22	1.09	0.15
A3	560	28.32	8.16	13.28	1.88	3.16	45.20	93.47	1.22	0.19

**Table 3:** Electrical properties of the CMZTSSe films annealed at different temperatures from 500 to 560 °C.

Sample	Temperature (°C)	Time (min)	ρ (Ω·cm)	n (cm^−3^)	μ (cm^−2^V^−1^s^−1^)	Conduction Type
A1	500	15	6.18 × 10^0^	9.22 × 10^17^	1.09 × 10^0^	p
A2	530	15	2.85 × 10^−1^	6.47 × 10^18^	3.31 × 10^−1^	p
A3	560	15	1.10 × 10^2^	5.54 × 10^17^	1.04 × 10^−1^	p

**Table 4:** The full width at half-maximum (FWHM), grain size, a-axis lattice constant (a), and c-axis lattice constant (c) for CMZTSSe films annealed for different times.

Sample	Temperature (°C)	Time (min)	a (Å)	c (Å)	Crystalline Size (nm)	FWHM
B1	530	10	5.665	11.334	52.8	0.190
B2	530	15	5.681	11.415	71.1	0.156
B3	530	20	5.684	11.453	61.8	0.171

**Table 5:** EDS results of the CMZTSSe films annealed for different times from 10 to 20 min.

Sample	Time (min)	Cu (at%)	Zn (at%)	Sn (at%)	Mg (at%)	S (at%)	Se (at%)	Se/(S + Se)	Cu/(Zn + Mg + Sn)	Mg/(Mg + Zn)
**B1**	10	29.66	9.88	11.60	2.39	10.23	36.24	77.99	1.24	0.19
**B2**	15	26.71	9.91	12.88	1.76	8.67	40.08	82.22	1.09	0.18
**B3**	20	29.81	8.49	11.43	1.18	2.26	46.44	95.36	1.41	0.12

**Table 6:** Electrical properties of the CMZTSSe films annealed for different times from 10 to 20 min.

Sample	Temperature (°C)	Time (min)	ρ (Ω·cm)	n (cm^−3^)	μ (cm^−2^V^−1^s^−1^)	Conduction Type
**B1**	530	10	6.18 × 10^0^	9.22 × 10^17^	1.09 × 10^0^	p
**B2**	530	15	2.85 × 10^−1^	6.47 × 10^18^	3.31 × 10^−1^	p
**B3**	530	20	1.10 × 10^2^	5.54 × 10^17^	1.04 × 10^−1^	p
